# Early Medical Practice in Bath

**Published:** 1964-10

**Authors:** Charles A. Marsh


### EARLY MEDICAL PRACTICE IN BATH

BY

CHARLES A. MARSH, M.D.

At the beginning of this century, Bath was a well-known spa and a residential town for those who had retired from service overseas. It was also known as a place that relied overmuch on past history, being associated with Roman remains, Bath chairs, and Bath buns. Cynics looked upon it as a sleepy old place, resting on its laurels. Actually this was far from being the case. Mineral water treatment was actively practised, and the general medical standard was at least as high as in comparable towns elsewhere. What many did not realize was that Bath had many other substantial interests; an engineering firm employing 1,000 hands, a number of smaller factories, and two main railways with their employees, all these activities maintaining the population of important suburbs.

The lapse of more than 60 years has naturally seen many changes in medical outlook. The span of life has increased by about 20 years. X-rays and radium have come in, the tuberculosis problem has been almost solved, thyroid and insulin treatment have been discovered, and what to my mind is the most spectacular advance of all, the outlook of a patient with pernicious anaemia has changed from 100% mortality to 100% viability. Unfortunately there is the other side of the picture. The improvement in the treatment of cancer is only slight, and for the common cold none at all.

At the beginning of the century antiseptic surgery was in full swing, aseptic methods following later. Rubber gloves were not used, and face masks were unknown. Preparation of surgeons' and dressers' hands was a great ceremony, and the magic letters "P.F.O." over a bed in a surgical ward meant that for 24 hours the preparation, especially of the skin at the site of operation, must be taken very seriously.

There was then no pathological department at Bath Hospital except for post mortems, and if I wanted a sputum tested for T.B. the patient had to pay for it. Blood counts were done privately, sedimentation rate tests had not come in, and measurement of the blood pressure was only beginning to be talked about. While I was at the London Hospital the physiology demonstrator, Leonard Hill, was busy inventing a sphygmomanometer. At first it consisted of a cocoa tin slit down the side, lined with an air-tight bag that was pumped up with a bicycle pump. Hill practised on students' arms, including my own, and the product came on the market as the Hill and Barnard sphygmomanometer. The Riva-Rocci instrument was more popular, but neither was used much in my early days of practice.

The Royal United Hospital was in the middle of the city, convenient for out-patients, but not otherwise suitable, and it has been moved to fine buildings at Upper Weston. The old building is now part of the Bath Technical College. The old hospital letter system was still in vogue; except in the case of emergencies, a patient wishing to attend was supposed to get a subscriber's letter. When I wanted to get a patient into hospital, this was rather a nuisance as the letter was not always available, but it was not very strictly insisted upon, and everyone concerned was relieved when the system was abolished. The hospitals were on a voluntary basis, except for the Fever Hospital which was run by the Corporation. Actually I had little difficulty in getting a bed for any patient who really needed it.

An ambulance service as we know it now simply did not exist. There was an ambulance nominally, but it was horse-drawn and so seldom used that the driver was full-time on other work. He had to be recalled from that work, get the horse out, and in due course attend. But it meant hours of delay, and the loss of half a day's work for the driver. For those who are used to the up-to-the-minute service of the present day, the difference is remarkable.

The first Baby Clinic was on a voluntary basis but sponsored by the Corporation. Its inception was partly due to a relative of my wife, and I was the first M.O. It was of considerable interest to one in general practice. Rickets was more prevalent at that time, and my observation was that it was due not only to bad feeding but also to absence of light, a large proportion of rickety babies coming from basement residence. Since the end of the first world war, the much extended clinics have been officered by the Public Health Service.

The system of District Nurses was fully developed in the early years of this century, as it continues to this day. But there were very few qualified midwives. Confinements were attended by a doctor with a so-called monthly nurse, or by a midwife of the Sarah Gamp type. The latter was no doubt sometimes a danger, but not always. I have in mind one who could neither read nor write, but those returns were always made in perfect copperplate writing, a daughter being the accessory. She let well alone, and I never knew one of her cases go wrong. Now, a large proportion of confinements are attended by midwives, and few by general practitioners. This was not so in early days; in my busiest years I did a lot of maternity work—about 100 deliveries a year, including those booked, those to which I was called by midwives, and miscarriages. Altogether I must have attended about a thousand births. When I go into a shop now it is quite common for the assistant while wrapping up the parcel to say "Do you remember bringing me into the world, Doctor?" My record was three forceps deliveries in 48 hours, and I once delivered triplets when doing a *locum* in Cornwall.

The old workhouse had only one medical officer, and he did not live on the premises. When the name was changed to Frome Road House, it was less unpopular, but it was still the one-night refuge for tramps, and the old buildings were unattractive. Large modern wards were built for military purposes during the second world war, and the name is now St. Martin's Hospital, a large modern institution, fully equipped and staffed.

The coming of war in 1914 had a profound effect on the medical profession, myself included; but as I am a Quaker with 300 years of quaker ancestry behind me, and as I was passed as fit for home service only, I stayed at home with the exception of a short period of voluntary service in France with the Friends Ambulance Unit. When the Americans were in Bath they built a large single-storey building which is now the Manor Hospital, a useful addition to Bath's hospital outfit.

Perhaps I may now recall some more autobiographical details bearing on medical matters. As a youth I was thin and fragile-looking, and my father, who was not a doctor, was doubtful whether I could stand the long medical training. He wanted an opinion other than that available from the local fraternity at Dorking, and took me to his fellow-Quaker Jonathan Hutchinson, then at the height of his career, and whom he knew a little. J. H. passed me, and as I am now in my 87th year his opinion seems to have been justified. None of my relatives being doctors, I do not know what gave my father the idea of making his son a doctor unless it was that his eldest daughter had married a cousin of Rickman Godlee, nephew and biographer of Lister.

So I entered the London Hospital for the Conjoint course in 1895, my father thinking that this would be less strenuous than taking the London University examinations. At that time most of the students at the London, other than those from Cambridge and Oxford, were taking the Conjoint course. The reason for this was that they had not passed the London University matriculation, at that time a terrible obstacle. When after a few weeks lecturers and others found that I had passed the dreaded "matric.", they intimated that I was several kinds of a fool not to go for the higher qualification. So I changed plans, and took both courses. Since then I understand that the terrible London Matric. has been brought more into line with that of other Universities.

Acquiring the M.R.C.S. and L.R.C.P. in February 1901, and the M.B. London in June 1901, I made a long journey to New Zealand and Fiji as ship's surgeon, and then had several post-graduate appointments. After this I wanted to get into practice but did not know how, as cash to buy a practice was not available. So I took locums for two years, mostly in south of England hoping, like Mr. Micawber, that something would turn up. Eventually it did. I had done a locum for a week in July 1905 for a doctor in Bath, and I heard from his representatives that he was mentally afflicted later in that year, and I was invited to come. The practice, never very large, had gone to pieces and was not worth much, but what was asked suited my pocket. It was very difficult then for an assistant to save enough to buy himself into practice; he got the princely sum of £100 a year, or if better favoured £110, with board and lodging. When he had met his personal expenses there was very little to save. Putting up a plate, otherwise known as "squatting", was not favoured in those days, but as I had a predecessor I did not incur that obloquy. I came to Bath in January 1906 in the throes of a general election which proved to be the "Liberal Landslide", and for the first time in its history Bath returned two Liberal members. I intended to stay for two years, and I have stayed fifty-eight.

At that time, in addition to the great Friendly Societies which provided medical attention as part of their benefit, there were what were called Slate Clubs, mostly run from public houses, and each had a contract with a doctor. My predecessor had a number of these that remained with me when most of the private patients had fled, but private patients soon began to come, and I tremble when I think of my audacity in becoming engaged to be married in August of my first year of practice. The universal rate of remuneration for a doctor taking Friendly Society or Slate Club patients was 4/– per annum for each one, on a list supplied in advance. Out of this the doctor had to supply medicine. He usually did his own dispensing, though if he had a large practice he might employ a dispenser. Expensive drugs could not be supplied, but I like to think that a patient's real needs were met, even if unreasonable requests were dealt with by what Lloyd George called "something coloured and something nasty". In those days the wage-earner was a "Club patient" and the rest of his family were private patients. A doctor took Club patients with a view to attending the dependents privately—good in theory but involving a load of bad debts, as will be appreciated from the fact that an engineer's labourer brought up a family on 22/– a week.

In my early days top hats, frock coats, and horses were beginning to go out, and motor cars to come in. It was not easy to keep a top hat on, in the open cars of that date. I used a pedal cycle at first, then a second-hand motor cycle which I spent more time on in repairs than in riding, then a second-hand car, and in 1911 the first new car, a Ford "Tin Lizzie". And what enjoyment we got from it! We could get to the Mendips in 45 minutes, and back in time for evening surgery. Also we took it to France twice, in 1912 and 1914, returning only just before the first war. In 1912 a car on a country road in France was enough of a curiosity for people to turn round and stare at it.

In 1911 a cloud appeared on the horizon. Lloyd George's Insurance Act began to be talked about and occupied first place in the political news until it came into force in 1913. It was looked upon with great suspicion. Many refused to "lick stamps". The medical profession which it so largely affected was gravely alarmed. Many, including myself, thought they were going to the workhouse. Lloyd George thought that as dispensing would be done by chemists, doctors should be content with what they used to receive from the Friendly Societies. This argument did not appeal in the slightest to the doctors, and fiery indignation meetings were held throughout the country. My own practice being almost entirely industrial, I was affected more than anyone else. Through taking part in the local meetings of the B.M.A. I was appointed Honorary Secretary of the Provisional Medical Committee, as it was first called, of the

Bath Division which at that time extended from Weston-super-Mare to just this side of Swindon. The name in due course was changed to Panel Committee, the personnel appointed under the Act being the same in Bath, though not in all areas. Many, perhaps most, doctors objected to the principle of having an important part of their equity controlled from outside, and it was ironical that those who objected most were those that were least affected. Many including myself objected to the inadequate terms, and at first I was a bitter opponent of the scheme. The opposition became so strong that Lloyd George became afraid that his whole scheme would fall through, and he unexpectedly raised his capitation fee by 2/6 a year. This does not sound much today, but in my case it changed the outlook from poverty to affluence. From being a strenuous opponent I became a strong advocate of the scheme, and sustained considerable criticism from the diehards. However, I lived it down, and within seven years was offered the chairmanship of the Bath Division of the B.M.A. which I accepted in 1924.

One has to remember a fundamental difference between the two insurance schemes. The first only included wage earners, the second included everybody. Private practice continued under the Act, now it is nearly washed out. The doctor now loses his private patients but is paid by the State on terms that would make the mouths of us older ones water. I well know that I shall be told that I forget how prices have risen, but when I recall that in 1906 a week's pay for a locum would about buy a suit of clothes, I think the comparison stands.

While Secretary of the local committees I used to attend the annual Panel Conferences in London, and was in close correspondence with headquarters of the B.M.A. whose Secretary, the late Dr. Alfred Cox appointed me one of the six witnesses for the B.M.A. on the Departmental Committee on excessive prescribing. When the first Act had come into force, my colleagues on the Panel Committee recognized my activities by the gift of a hall clock, which still merrily chimes the quarters. When I retired from the Secretaryship after 13 years there came another presentation, this time of old prints of Bath.

In addition to the long voyage to New Zealand already mentioned, I have always been fond of travel. When working hard I made a point of taking one good holiday a year, leaving a locum in charge. At first the distances were short, chiefly to Switzerland; but later having taken Dr. Burke Cuppage into partnership I was able to get away for longer, as the following list of places visited will show:—the summit of Mont Blanc, Cape Horn, Timbuctoo, the Amazon, Kenya, Uganda, the Sudan, Trinidad, Iceland, Egypt and the North African countries, most of the European countries, and Ethiopia as the guest of the Emperor.

Chess is another hobby, and I have been President of the Somerset County Chess Association and of the Bath Chess Club for more than 20 years, and still am. Being impressed by the harm done by alcoholism I have been "teetotal" all my life, though I believe I have attended more publicans than any other doctor in Bath.

Looking back on my long life, I remember that as a boy I wanted to be an electrical engineer while my father wanted me to be a doctor. He had his way, and I am profoundly thankful, for I have retained electricity as a hobby; and in spite of the tinkle of the night bell and other drawbacks, the net result is a feeling of satisfaction in a life of medical and social activities, and gratitude to those who have helped me to attain it.

